# Bliss' and Loewe's additive and synergistic effects in *Plasmodium falciparum* growth inhibition by AMA1-RON2L, RH5, RIPR and CyRPA antibody combinations

**DOI:** 10.1038/s41598-020-67877-8

**Published:** 2020-07-16

**Authors:** Yvonne Azasi, Shannon K. Gallagher, Ababacar Diouf, Rebecca A. Dabbs, Jing Jin, Syed Yusuf Mian, David L. Narum, Carole A. Long, Deepak Gaur, Simon J. Draper, Michael P. Fay, Louis H. Miller, Kazutoyo Miura

**Affiliations:** 1 Laboratory of Malaria and Vector Research, National Institute of Allergy and Infectious Diseases, National Institutes of Health, 12735 Twinbrook Parkway, Rockville, MD 20852 USA; 2 Biostatistics Research Branch, National Institute of Allergy and Infectious Diseases, National Institutes of Health, Rockville, MD USA; 3 The Jenner Institute, University of Oxford, Oxford, UK; 4 Laboratory of Malaria and Vaccine Research, School of Biotechnology, Jawaharlal Nehru University, New Delhi, India; 5 Laboratory of Malaria Immunology and Vaccinology, National Institute of Allergy and Infectious Diseases, National Institutes of Health, Rockville, MD USA

**Keywords:** Parasitology, Vaccines

## Abstract

Plasmodium invasion of red blood cells involves malaria proteins, such as reticulocyte-binding protein homolog 5 (RH5), RH5 interacting protein (RIPR), cysteine-rich protective antigen (CyRPA), apical membrane antigen 1 (AMA1) and rhoptry neck protein 2 (RON2), all of which are blood-stage malaria vaccine candidates. So far, vaccines containing AMA1 alone have been unsuccessful in clinical trials. However, immunization with AMA1 bound with RON2L (AMA1-RON2L) induces better protection against *P. falciparum* malaria in *Aotus* monkeys. We therefore sought to determine whether combinations of RH5, RIPR, CyRPA and AMA1-RON2L antibodies improve their biological activities and sought to develop a robust method for determination of synergy or additivity in antibody combinations. Rabbit antibodies against AMA1-RON2L, RH5, RIPR or CyRPA were tested either alone or in combinations in *P. falciparum* growth inhibition assay to determine Bliss' and Loewe's additivities. The AMA1-RON2L/RH5 combination consistently demonstrated an additive effect while the CyRPA/RIPR combination showed a modest synergistic effect with Hewlett’s $S=1.07 \left[95\% \mathrm{C}\mathrm{I}: 1.03, 1.19\right].$ Additionally, we provide a publicly-available, online tool to aid researchers in analyzing and planning their own synergy experiments. This study supports future blood-stage vaccine development by providing a solid methodology to evaluate additive and/or synergistic (or antagonistic) effect of vaccine-induced antibodies.

## Introduction

Malaria remains a global health problem with over 200 million cases and more than 400,000 deaths annually^1^. Most of these deaths are caused by the most virulent parasite *Plasmodium falciparum*. Ongoing interventions with insecticides, bed nets and artemisinin combination therapy had led to a decline of mortality and morbidity; however, the decline has stalled in recent years. It is still the hope that a malaria vaccine will facilitate the much-needed step towards eradication of the disease, especially in Sub-Saharan Africa where it is most relevant. There are many malaria vaccine candidates in development; these are targeted at the pre-erythrocytic-stage, blood-stage, or transmission-stage of the parasite. The most advanced malaria vaccine is the RTS,S/AS01 vaccine which targets the parasite's circumsporozoite protein at the pre-erythrocytic stage. In a Phase III trial with RTS,S/AS01, the vaccine reduced clinical malaria cases by 39% and severe cases by 26% in children^2^. Therefore, on-going efforts are focused on the development of more effective next-generation vaccine candidates.

Clinical manifestations of the disease occur in the blood-stage infection of the parasite's lifecycle, where the parasite invades the host red blood cells (RBC), multiplies and invades other RBC to continue the asexual cycle. Invasion of the RBC by merozoites involves: (i) the initial contact by the merozoite, (ii) reorientation and deformation, (iii) binding of the merozoite to the RBC, (iv) formation of a moving junction, (v) internalization of the merozoite, and (vi) resealing the parasitophorous vacuole^3–5^. Various antigens such as merozoite surface proteins (MSP) 1^6^ and 3^7^, erythrocyte binding antigen-175^8^, apical membrane antigen 1 (AMA1)^9^ and reticulocyte-binding protein Homolog 5 (RH5)^10^ are involved in these steps and have been the focus of asexual blood stage vaccine development.

AMA1 was a promising vaccine candidate since it elicited biologically active antibodies (as measured in vitro) following human vaccinations^11–13^. However, an AMA1 vaccination did not lead to protection in Controlled Human Malaria Infection (CHMI) model with a homologous clone^14^. In addition, AMA1 is highly polymorphic and that may be an another reason why vaccination of individuals with one or two-allelic forms of AMA1 did not protect against clinical disease in Phase IIa or IIb trials^14–18^. Therefore, further improvement in AMA1-based vaccines has been awaited. AMA1 binds to the rhoptry neck protein, RON2, to form a moving junction during merozoite invasion of erythrocytes. In a preclinical trial, vaccination of *Aotus* monkeys with the AMA1-RON2L complex completely protected 50% of the monkeys and delayed blood-stage infection in 75% of the remaining animals against homologous *P. falciparum* challenge, while vaccination with AMA1 alone only partially protected 13% of the monkeys^19^. Thus, AMA1 in complex with its rhoptry binding partner appears to be a more potent vaccine candidate. Another current leading blood-stage vaccine candidate is the rhoptry protein, RH5. RH5 is essential for binding to the host erythrocyte receptor basigin to facilitate invasion^20^. RH5 forms a complex with RH5-interacting protein (RIRP)^21^ and cysteine-rich protective antigen (CyRPA)^22^, and recent data suggest the whole RH5/RIPR/CyRPA complex can also bind to the RBC^23^. Vaccination of *Aotus* monkeys with RH5 protein/adjuvant showed complete protection against blood-stage infection in 33% of the monkeys while the rest cleared the infection with no treatment^24^. Also, in a Phase Ia clinical trial in healthy UK adults, RH5 vaccination induced significantly higher RH5 antibody responses than those observed in naturally-exposed individuals in malaria endemic regions, and the vaccine-induced antibodies showed biological activity as judged by the in vitro growth inhibition assay (GIA)^25^. Like AMA1-RON2L or RH5 antibodies, antibodies to CyRPA and RIPR (the other members of the RH5 complex) have parasite growth inhibitory activity in animal immunization studies^21,22^. RH5 antibody has been tested in combination with CyRPA, RIPR or AMA1 antibodies among others, and studies of antibody combinations of AMA1 or the RH5 complex components and with other antigens are summarized in Table 1. While an additive growth inhibitory effect was observed with RH5/AMA1^26^ and RH5/RIPR antibody combinations^22^, combination with AMA1-RON2L antibodies has not been evaluated. In this report, we evaluated antibody combinations of RH5, RIPR or CyRPA with AMA1-RON2L in GIA with the aim of finding other antigens that may act additively or synergistically to improve the efficacy of the AMA1-RON2L vaccine candidate.

**Table 1:** Antibody combination studies involving the RH5 complex (RH5, CyRPA and RIPR) or AMA1.

Antibody combination	Method	Study reference
AMA1 with GAMA	Bliss’	Arumugam et al. 2011^38^
RIPR with EBA175, RH2a/b or RH4.9; a mixture of all 4	NA	Chen et al. 2011^21^
RH5 with EBA175	Bliss’	Ord et al. 2012^39^
RH5 with AMA1, RH4, MSP1, RAP3, RH2, EBA175 or Pf38	Bliss’ and Loewe’s	Williams et al. 2012^26^
CyRPA with RH5 RIPR with RH5	Bliss’	Reddy et al. 2015^22^
CyRPA with RH5, EBA181, MSRP5, SERA9 or RAMA RH5 with RAMA, MSRP5, EBA181 or SERA9	Loewe’s	Bustamante et al. 2017^40^
CyRPA-RH5	Bliss’	Favuzza et al. 2017^41^
RH5 with AMA1, CyRPA, RIPR, MSP1 or RH4	Bliss’	Alanine et al. 2019^34^
CyRPA with RH5	Bliss’	Illingworth et al. 2019^42^
CyRPA with RIPR or RH5 RIPR with RH5 CyRPA with RIPR and RH5	Bliss’	Healer et al. 2019^28^

N.A.: Neither Bliss' nor Loewe’s additivity can be determined from the published results.

Performing GIA with combinations of multiple antibodies has been done previously but, in some cases, different or contrasting results have been reported. For example, antibody combinations of RH5 with CyRPA were reported to exhibit a variation in their ability to induce synergistic or additive inhibition of parasite growth^19,24,25^. Such variability could be explained, at least in part, due to differences in the recombinant protein or adenoviral based vaccines used to induce the antibodies, antibody concentrations in the experiments, animal species where antibodies were raised, and/or by methods for analysis.

There are two common, but different, definitions of additivity and synergy used to evaluate the effect of antibody combinations: Bliss’ and Loewe’s^27^. Bliss’ synergy may be estimated for specific doses with few concentrations tested but Bliss’ synergy may in certain cases define an antibody to be synergistic with itself (discussed in detail later). Loewe’s synergy avoids this “self-synergy” problem, but it is more difficult to estimate.

In this study, we examined four combinations of antibodies (RH5/AMA1-RON2L, CyRPA/AMA1-RON2L, RIPR/AMA1-RON2L and CyRPA/RIPR) using Bliss' model first, then selected two combinations (RH5/AMA1-RON2L and CyRPA/RIPR) which were further evaluated using Loewe's model. We offer a robust statistical method to determine Loewe’s synergy or additivity of antibody combinations in GIA. Furthermore, the new analysis can be performed by an online tool which we provide here.

## Results and discussion

**Figure 1:**
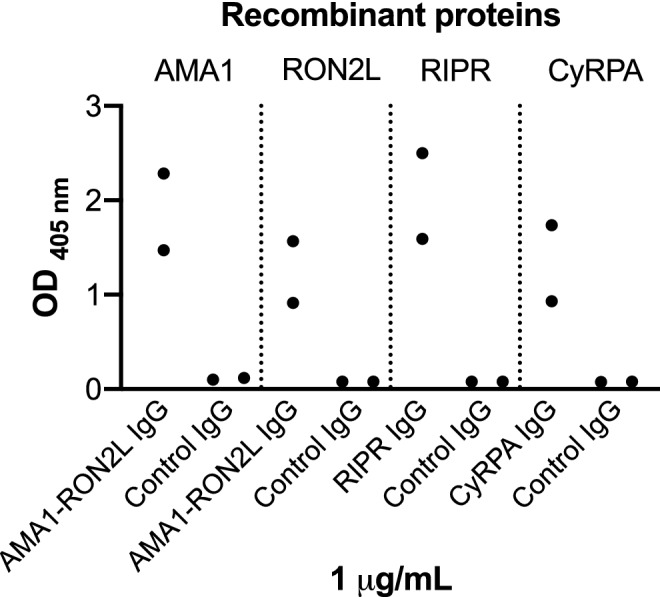
Total IgGs from rabbits immunized with AMA1-RON2L, RIPR or CyRPA binds to their specific antigens. Antibody reactivity of 1 μg/mL of AMA1-RON2L, RIPR, CyRPA or control total IgG (two rabbit IgGs per antigen) to 1 μg/mL of recombinant AMA1, RON2 peptide, RIPR or CyRPA protein was tested in ELISA. Each dot represents the average OD value of each rabbit IgGs from two independent assays. RH5 antibody was not tested by ELISA as the IgG came from a previously published study^26^.

**Figure 2:**
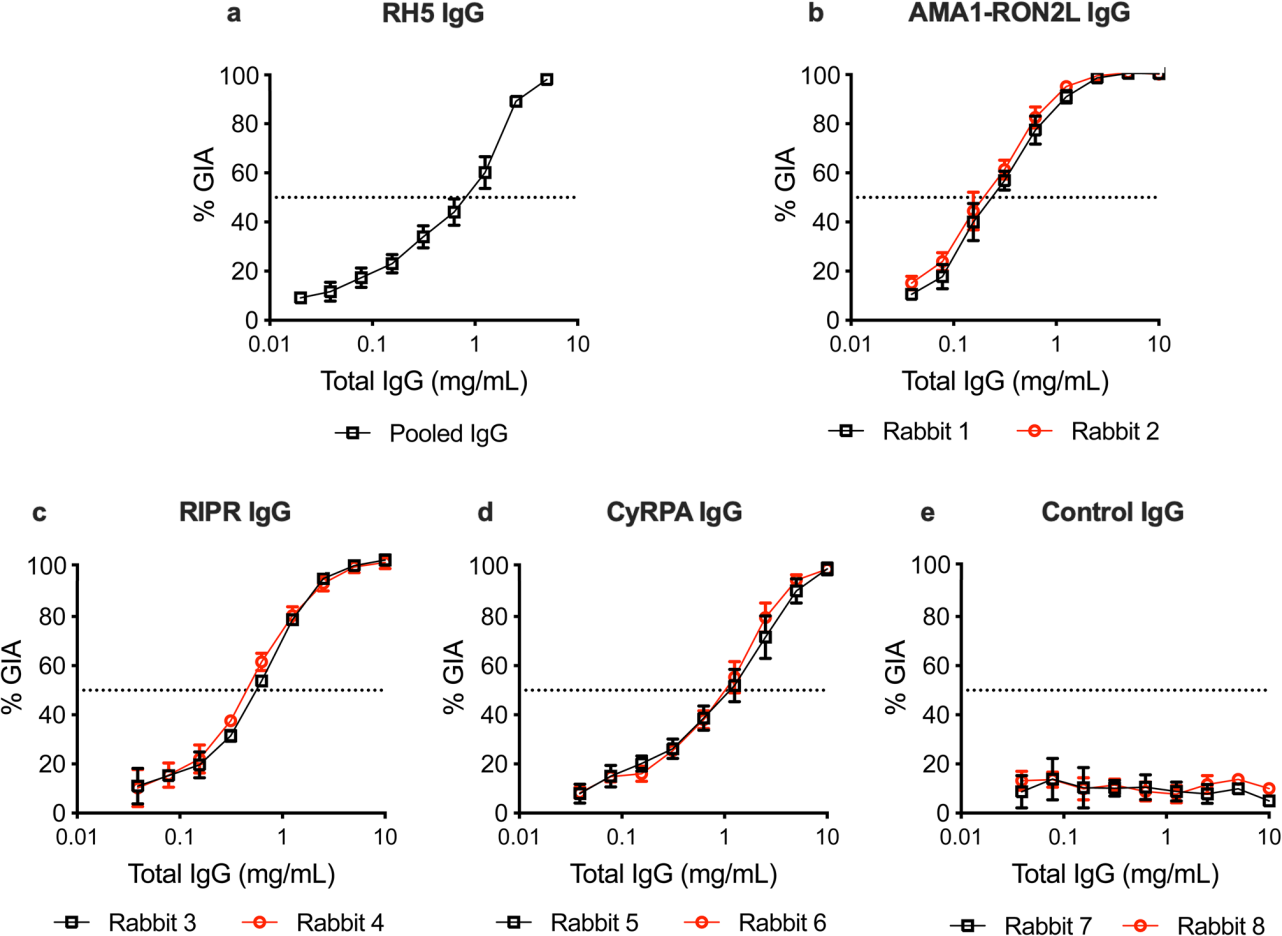
Growth inhibition activity of AMA1-RON2L, RIPR, CyRPA and RH5 antibodies. Increasing concentrations of purified total IgG from rabbits immunized with RH5 **(a)**, AMA1-RON2L **(b)**, RIPR **(c)**, CyRPA **(d)** or Control IgG **(e)** were tested for growth inhibition of 3D7 clone parasites. Data shown are the mean and SEM of at least two independent experiments each with three replicate wells. Dashed line shows 50% inhibition. For RH5 **(a)**, GIA was done with a pool of total IgG from rabbits immunized with RH5. For the other antigens or negative control **(b–e)**, two total IgG samples from two rabbits were tested individually (two lines per antigen).

To conduct GIA with mixtures of antibodies targeting two different antigens, antibodies were made against each of the AMA1-RON2L, RIPR and CyRPA antigens in rabbits, and all the antibodies reacted with their respective antigens in ELISA as shown in Fig. 1. The antibodies had a parasite growth inhibitory effect as expected, with AMA1-RON2L antibody being the most potent in terms of total rabbit IgG, followed by RIPR and CyRPA antibodies (Fig. 2). Antibodies from each of the two rabbits immunized per group (AMA1-RON2L, CyRPA and RIPR IgG) showed identical inhibitory activity at the same total IgG concentration, therefore, purified total IgG from one rabbit per group was used in the antibody combinations. RH5 antibodies used in this study were a pool of purified total IgG from five immunized rabbits and generated in a previous study^26^ where the five individual RH5 purified IgGs demonstrated the same activity at the same RH5-specific concentration. We confirmed the biological activity of the pooled RH5 IgG (Fig. 2a) before performing the antibody combination assays.

### Bliss’ additivity assessment of RH5/AMA1-RON2L, RIPR/AMA1-RON2L, CyRPA/AMA1-RON2L and CyRPA/RIPR antibody combinations

**Figure 3:**
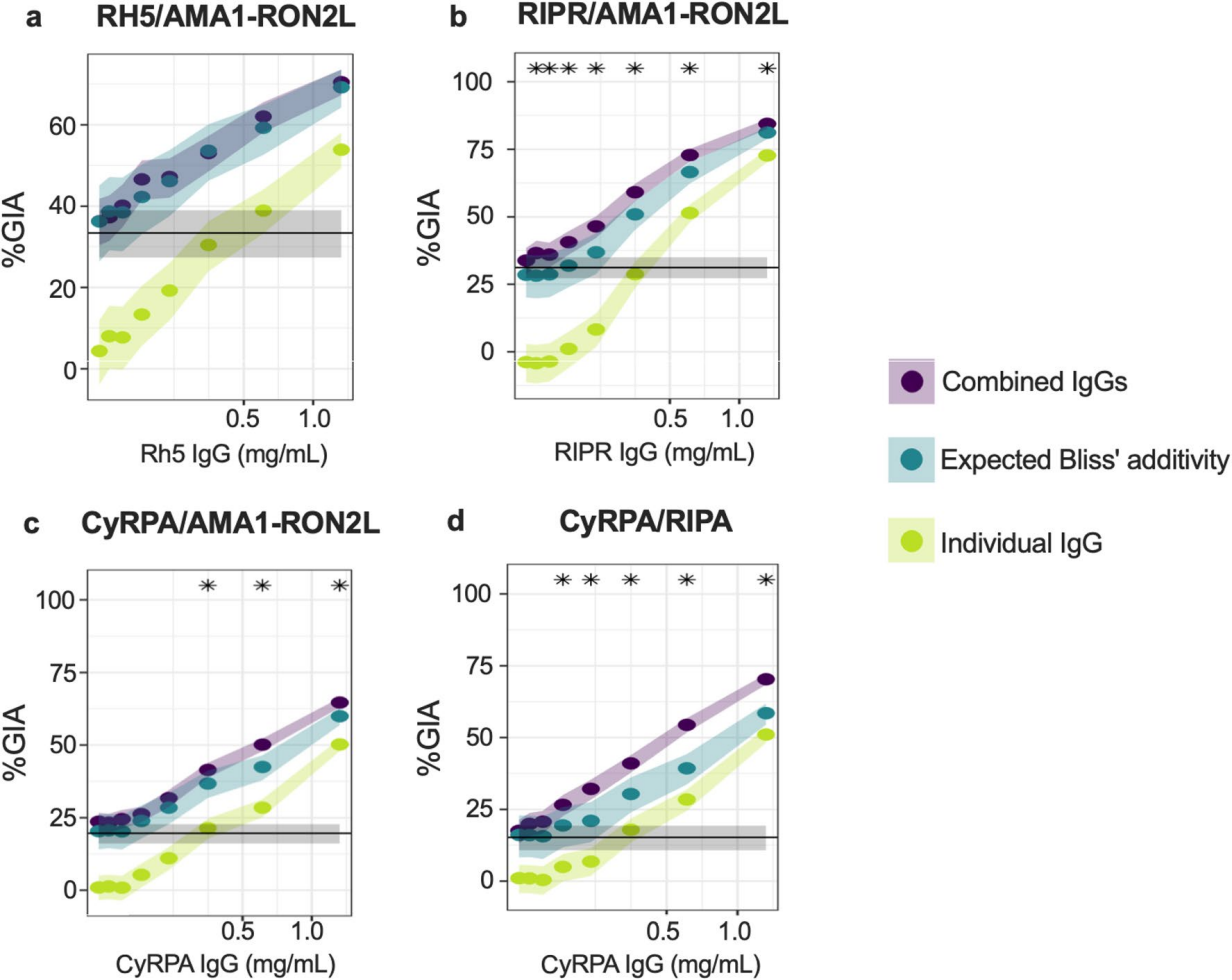
Growth inhibition activity of RH5/AMA1-RON2L, RIPR/AMA1-RON2L, CyRPA/AMA1-RON2L or CyRPA/RIPR total IgGs combinations. A fixed concentration (0.078 mg/mL) of AMA1-RON2L antibody was mixed with various concentrations of RH5 **(a)**, RIPR **(b)** or CyRPA **(c)** antibodies. For the CyRPA/RIPR combination **(d)**, a fixed concentration of RIPR antibody was tested with various concentrations of CyRPA antibody. Data shown are from n ≥ 2 independent experiments, each with 2 or 3 replicate wells. % GIA (y-axis) means % inhibition determined by GIA with 3D7 clone. Shaded regions are 95% confidence intervals (95%CI) by the parametric bootstrap percentile method. The green-yellow band shows % GIA of the titrated IgG: RH5 **(a)**, RIPR **(b)** or CyRPA (**c** and **d**), which was tested alone at the indicated concentrations; blue shows % GIA from the Bliss model assuming Bliss’ additivity (no interaction effect); and purple shows experimentally observed (plus 95%CI) % GIA from the mixture of the two IgGs. The grey band shows % GIA from the fixed concentration of the fixed IgG when tested alone; AMA1-RON2L **(a–c)** or RIPR **(d)**.

A fixed concentration of AMA1-RON2L antibody was mixed with various concentrations of RH5, RIPR or CyRPA antibodies to determine Bliss’ additivity. The CyRPA/RIPR combination (fixed dose of RIPR antibody with various concentrations of CyRPA antibody) was also included because synergy between CyRPA and RIPR monoclonal antibodies in inhibiting parasite growth has been reported^28^. The results of the Bliss’ additivity experiments are shown in Fig. 3. When an observed inhibition for the combination (purple) was significantly higher than Bliss' independent activity (blue), it was considered as synergy between the two antibodies (indicated with asterisks in Fig. 3). The RH5/AMA1-RON2L antibody combination had an additive growth inhibitory activity (Fig. 3a), while the other combinations had either additive (at lower concentrations of RIPR or CyRPA antibodies in Fig. 3c, d ) or otherwise synergistic inhibitory activity (Fig. 3b–d).

Having obtained these results, it is important to note that Bliss’ additivity and Loewe’s additivity are two different ways of defining additivity (and hence of defining synergy and antagonism). Bliss’ additivity is commonly used in this field to assess interaction effects between antibodies (Table 1) and has a clear interpretation and visualization. In addition, there is a practical benefit to perform the Bliss’ additivity test. Given a minimum of three experimental conditions (antibody A alone, B alone and combination of A and B) can determine Bliss’ additivity. This saves time, effort and test materials as compared to the Loewe’s additivity test. These reasons are why we used the Bliss’ additivity model first to screen for possible synergistic pairs of antibodies. However, determination of synergy by Bliss’ additivity has a major disadvantage in that it can fail the “sham” thought experiment, depending on the shape of the dose response curve and/or test concentration of antibodies. The "sham" experiment states that if the two concentrations of the same antibody A are mixed (and we have sufficient replicates that we can ignore the GIA assay measurement variability), then the mixture should show additivity, because the same antibody A should not negatively or positively interact with itself. For example, if 1 mg/mL of A gives 25% GIA, and 2 mg/mL of A gives 50% GIA, then the Bliss model predicts 62.5% GIA at 3 mg/mL (i.e., (1 − (1 − 0.25) × (1 − 0.5) ) × 100 = 62.5). However, based on the dose–response curve of A, even when there is no GIA assay variability, it is possible that we might see either Bliss’ synergy (i.e., 3 mg/mL of A shows > 62.5% GIA) or Bliss’ antagonism (i.e., < 62.5% GIA at 3 mg/mL) when looking at a dose pair of A and A. As a consequence, we sought a different definition of additivity to prevent “sham” experiments from implying either synergy or antagonism. Another problem of using the Bliss’ additivity model is that the conclusion could be changed depending on the antibody concentration used. As seen in Fig. 3c and d, combinations with lower concentrations of CyRPA antibodies showed additive effects, whilst the same combination, with higher concentrations of CyRPA antibodies, demonstrated synergy effects. A similar dose–effect on the additive versus synergistic conclusion has been reported in previous studies, where multiple dose combinations were examined using the Bliss model^22,26^.

### Loewe’s additivity assessment of RH5/AMA1-RON2L and CyRPA/RIPR antibody combinations

Loewe’s additivity is defined so that the "sham" thought experiment will never allow a substance to be synergistic with itself; it will always be additive with itself. In addition, with a Loewe's additivity model, (essentially) a single parameter, Hewlett’s *S* is calculated using all dose data and this statistic indicates whether there is Loewe’s synergy, antagonism or additivity. On the other hand, however, Loewe's model has a disadvantage; it generally requires more data points to determine the effect as compared to the Bliss’ additivity model. Therefore, out of the four combinations tested for Bliss' additivity, only two selected combinations were further evaluated whereby each antibody was tested at 6 different concentrations (including 0 µg/mL); i.e. a total of 36 combinations (it is called "6 × 6 grid" in this manuscript) per assay, and two independent assays, or biological replicates, were performed for each combination. One of the selected combinations was RH5/AMA1-RON2L, which showed additive effects at all concentrations tested in the Bliss’ additivity analysis (Fig. 3a), and the other combination was CyRPA/RIPR, which showed the largest difference between observed inhibitions and Bliss’ predicted additive values (Fig. 3d).

**Figure 4:**
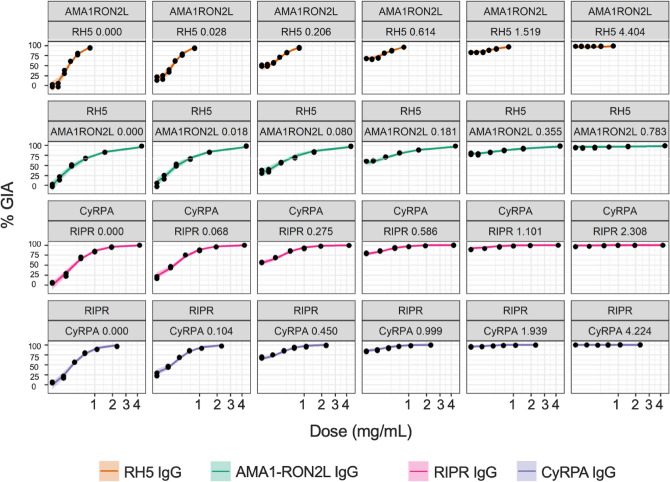
Model fits. The median estimate % GIA (color lines) and pointwise 95% central quantiles (color ribbons) are shown with actual observed % GIA (black dots). The top line in each row refers to the titrated antibody (the test concentration is shown in x-axis) and the second line in each row is the antibody that is being fixed to a certain quantity (the concentration in mg/mL is shown next to the antibody name). The top two rows show the data of RH5/AMA1-RON2L combination, and the bottom rows for the CyRPA/RIPR combination.

**Figure 5:**
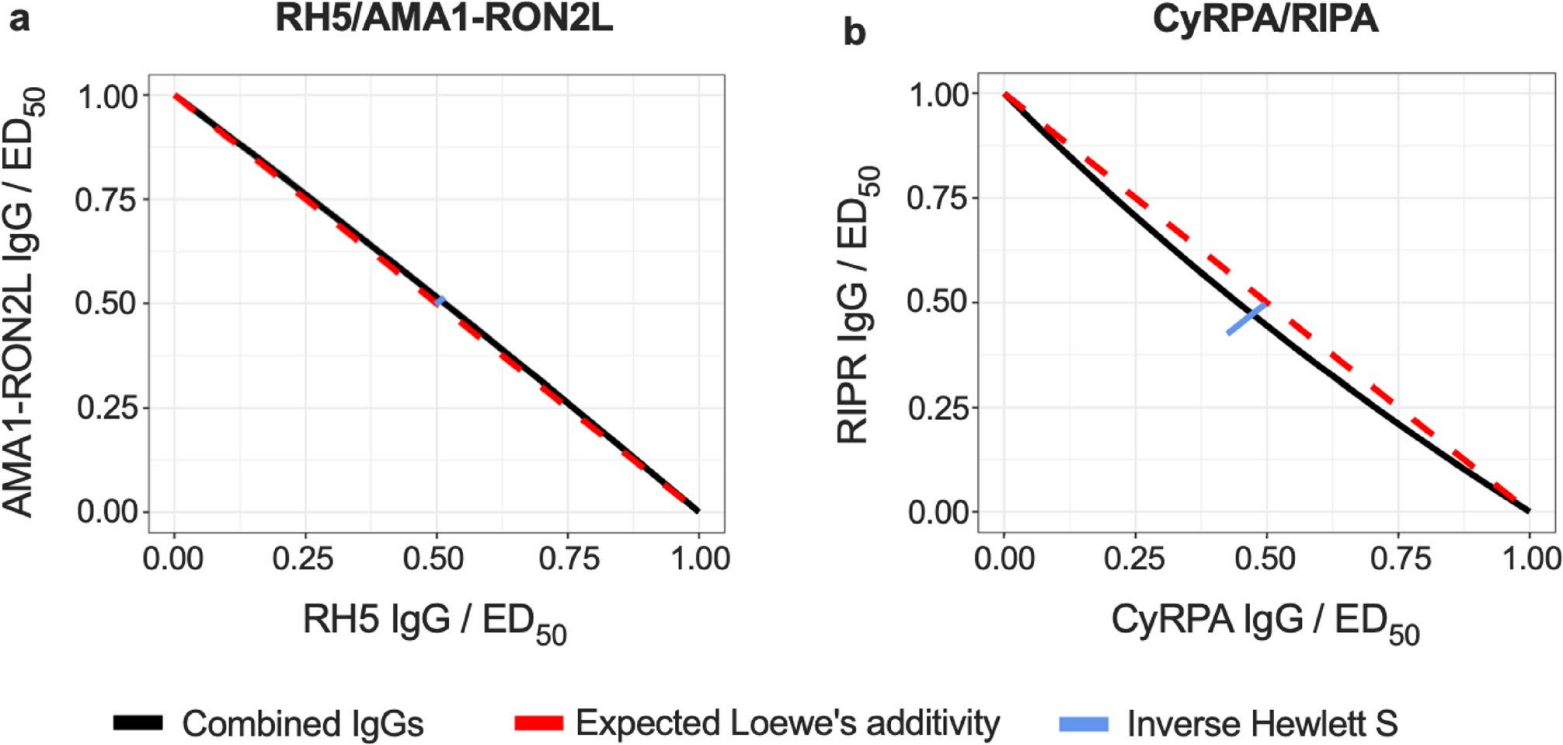
Isobolograms for model fits. The ED50 line for observed dose-combinations (black) and the 95% confidence interval of the inverse Hewlett *S* statistic (blue line), which corresponds to equal doses (after scaling by the ED50) of the two concentrations are shown; **(a)** RH5/AMA1-RON2L combination, and **(b)** CyRPA/RIPR combination. Dashed red line shows null synergy/antagonism interaction, and the synergy/antagonism hypothesis is rejected when an inverse Hewlett S line intersects with the null line.

We next developed a new Loewe’s additivity model as shown in Eqs. (2) and (3), and determined the best-fit parameters for each combination as shown in Table 2. The expected model fit, and 95% CI are plotted in Fig. 4. Since most of the observed values were contained within the 95% CI regions, the model is considered to fit the data well. Based on the Loewe’s model, there was no significant synergy effect for the pair of RH5/AMA1-RON2L with ${\tau }_{1}=$-0.06 (95% CI: [− 0.11, 0.01]) and Hewlett’s S = 0.986 (95% CI: [0.968, 1.002]); therefore, we cannot reject the null hypothesis that the combination is additive (Tables 2 and 3). This result was similar to what was previously reported with another RH5 and AMA1 antibody combination^26^. On the other hand, the CyRPA/RIPR combination had a significant, although modest, synergy effect with ${\tau }_{1}=0.25$ (95% CI: [0.02, 0.84]) and Hewlett’s S = 1.066 (95% CI: [1.025, 1.192]). The isobolograms, which show the effect of synergy for the two pairs of antibodies are seen in Fig. 5. For the RH5/AMA1-RON2L combination (Fig. 5a), the predicted ED50 curve almost completely overlapped with the dashed red line indicating additivity, while for CyRPA/RIPR (Fig. 5b), the predicted ED50 curve fell below the dashed red line, indicating synergy.

**Table 2:** Parameter estimates from model.

Parameter	Mean	2.5 quantile	97.5 quantile
RH5 ED50 $({\upbeta }_{\mathrm{A}})$	0.25	0.215	0.28
RH5 shape (${\upgamma }_{\mathrm{A}}$)	0.53	0.47	0.57
AMA1RON2 ED50 $({\upbeta }_{\mathrm{B}})$	0.15	0.13	0.16
AMA1RON2 shape (${\upgamma }_{\mathrm{B}}$)	0.92	0.83	1.03
Interaction RH5-AMA1RON2 (${\uptau }_{1\mathrm{A}\mathrm{B}}$ )	− 0.06	− 0.11	0.01
Shape RH5-AMA1RON2 (${\uptau }_{2\mathrm{A}\mathrm{B}})$	0.10	− 4.6	4.99

Parameter	Mean	2.5 quantile	97.5 quantile
CyRPA ED50 $({\upbeta }_{\mathrm{C}})$	0.25	0.22	0.29
CyRPA shape (${\upgamma }_{\mathrm{C}}$)	0.74	0.67	0.85
RIPR ED50 $({\upbeta }_{\mathrm{D}})$	0.22	0.19	0.25
RIPR shape (${\upgamma }_{\mathrm{D}}$)	0.81	0.71	0.91
Interaction CyRPA-RIPR (${\uptau }_{1\mathrm{C}\mathrm{D}}$ )	0.25	0.02	0.84
Shape CyRPA-RIPR (${\uptau }_{2\mathrm{C}\mathrm{D}})$	− 0.13	− 1.77	4.35

Parameter estimates for each of the experiments RH5/AMA1-RON2L and CyRPA/RIPR**. **The mean estimate and 2.5 and 97.5 quantiles for each parameter (which stands in for a 95% confidence interval) are shown. The parameter estimates correspond to the parameters shown in Eq. (3).

**Table 3:** Table of Hewlett *S* statistics.

Antibody A	Antibody B	Mean S	2.5% S	50% S	97.5% S
RH5	AMA1RON2	0.986	0.968	0.984	1.002
CyRPA	RIPR	1.066	1.025	1.069	1.192

Hewlett S statistic mean, median, and 2.5 and 97.5 quantiles from bootstrap replications for both combinations are shown.

The combination of AMA1-RON2L antibody with RH5 antibody resulted in an additive effect (Figs. 3a and 5a). The step of RH5 binding to basigin on RBC during invasion process is described to precede junction formation by AMA1 binding to RON2^29^. In other words, the inhibition mechanisms for those two antibodies are likely to be independent, and the independency may explain the additive inhibition seen for the combination. On the other hand, the combination of CyRPA and RIPR antibodies demonstrated a synergistic effect (Figs. 3d and 5b), which might be due to the similar inhibition mechanisms of the two antibodies. CyRPA serves as the contact between RH5 and RIPR in the complex for efficient RH5 binding to the erythrocyte^23^, therefore, the CyRPA and RIPR antibodies may act together. Further study is required to uncover the mechanism(s) why a certain combination of antibodies shows an additive or synergistic effect. Worthy of note, an "additive" antigen is not necessarily to be excluded from a first choice of vaccine candidate. The antigen selection needs to consider multiple aspects, such as biological activity of antibodies induced by each antigen, cost of antigen production and polymorphism in the target molecules. The precise evaluation for an additive and synergistic effect will support a rational antigen selection.

### Simulations and online apps for Loewe’s additivity assessment

To support future studies where researchers assess in vitro antibody combinations to evaluate additive and/or synergistic (or antagonistic) effect of antibodies, additional simulations were performed using GIA data obtained from this study. In addition, we used published GIA data of RH5/RH4 antibody combinations which were reported in a previous study^26^; here this particular combination demonstrated a strong Loewe's synergistic effect (although to note, the GIA data were analyzed differently in the previous study). As expected from the previous report, the RH5/RH4 combination analysed by our Loewe’s additivity model showed a strong synergistic effect with ${\tau }_{1}=$ 35.76 (95% CI: [23.54, 61]) and the corresponding Hewlett $S$ is $\widehat{S}$= 3.53 (95% CI: [2.92, 4.37]). While both analyses gave the same conclusion, our Loewe’s additivity model has the advantage of using a functional form to leverage all available data to determine the synergy effect as opposed to extrapolating a contour line. The best-fit parameters and the isobolograms for the RH5/RH4 combination from our Loewe’s additivity model are shown in the Supplementary Material.

**Figure 6:**
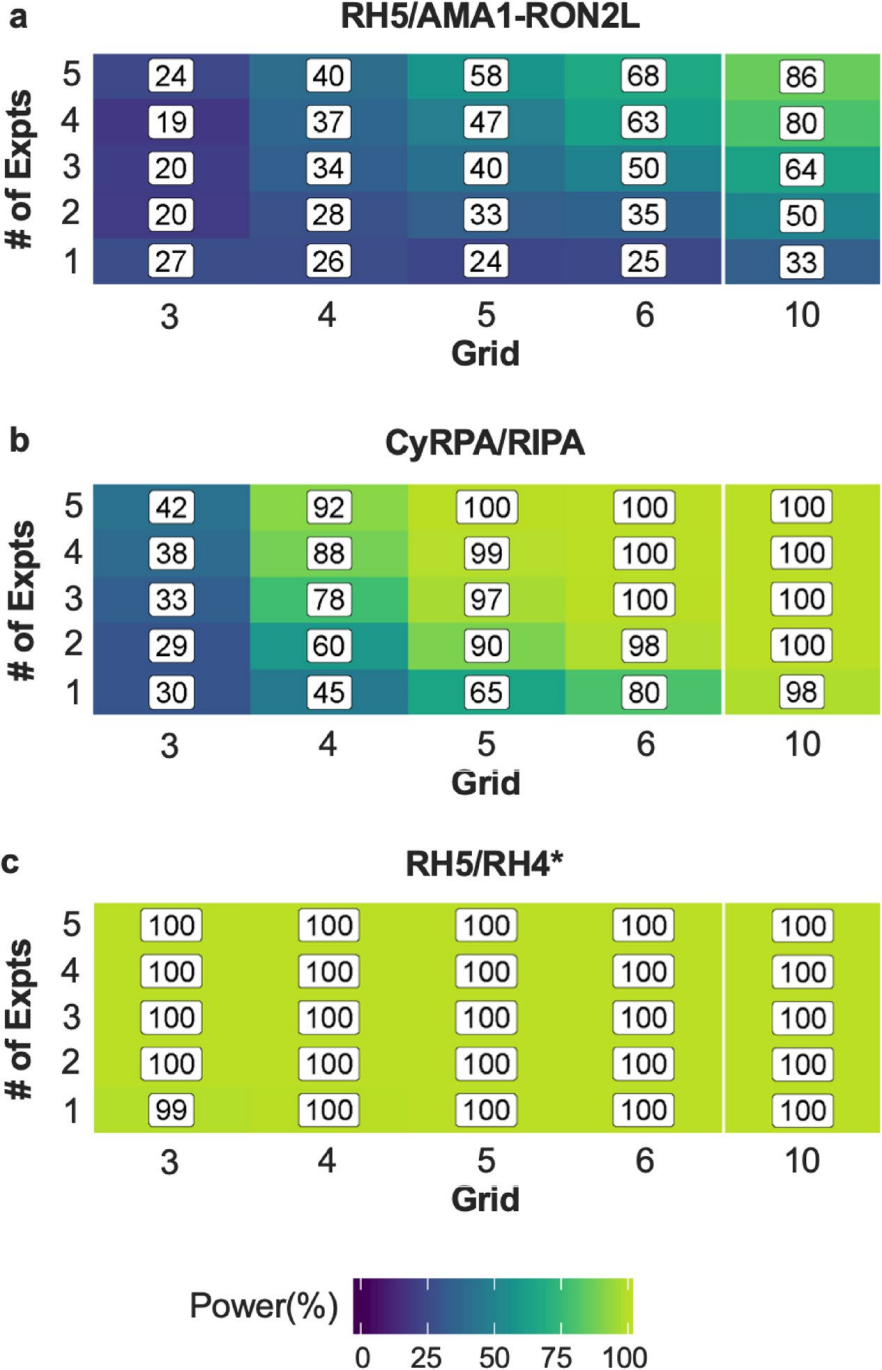
Power simulation in three scenarios. The power (%) of our model to detect significant interactions depending on the initial parameters are shown. Three sets of estimated initial parameters corresponding to the model estimates from the pairs of RH5/AMA1-RON2L **(a)**, CyRPA/RIPR **(b)**, and RH5/RH4 (**c**, *data from Williams et al*.*^26^). **(a)** has an interaction effect of close to zero, (${\tau }_{1} =$ − 0.06), **(b)** has a modest synergy effect (${\tau }_{1}=$ 0.25), and **(c)** has a large synergy effect (${\tau }_{1}=$ 36). The *x*-axis is the number of concentrations for one of the antibodies tested in an assay. For example, grid of 3 means each antibody is tested at 3 different concentrations, totaling 3 × 3 = 9 conditions per assay. The *y*-axis is the number of times to repeat the assay.

With respect to our new model of Loewe’s synergy, simulations showed using a parametric bootstrap analysis that the percent of false positives, i.e. finding a synergy (or antagonism) when there was none, was less than or equal to the specified $\alpha$-level. Also, the power to detect a significant interaction (either synergistic or antagonistic) was calculated in different test conditions using three different antibody combinations. In the simulations, the best-fit parameters were fixed based on the RH5/AMA1-RON2L, CyRPA/RIPR or RH5/RH4 combinations (the best-fit parameters are shown in Table 2 and Supplementary Material), then various test conditions in terms of their grid sizes (e.g. whether each antibody was tested at two different concentrations, i.e. 2 × 2 grid, or at three concentrations, 3 × 3 grid) and number of repeat (independent) assays were evaluated. For example, Fig. 6a shows the simulation results using the best-fit parameters (β_A_, β_B_, γ_A_, γ_B_, τ_1_ and τ_2_) calculated from the RH5/AMA1-RON2L combination data, and determined the power to detect a significant antagonistic effect if true τ_1_ is equal to -0.06. When each antibody was tested at 6 different concentrations (6 × 6 grid) and the assay was repeated two times, the study design had only 35.2% power to detect a significant antagonism. The simulations with the three different antibody combinations showed the power to detect a significant effect is quite sensitive to the magnitude of ${\tau }_{1},$ the primary interaction effect (Fig. 6). In order to detect the small value of antagonism ${\tau }_{1}= - 0.06$. (Fig. 6a), at least 5 repeat experiments on an evenly spaced 10 × 10 grid of dose combinations, totaling 500 observations (100 points per assay × 5 assays) are required to have a > 80% chance of finding a significant antagonistic effect. However, in the case of the CyRPA/RIPR antibody combination with ${\tau }_{1}=0.25$, an evenly spaced 4 × 4 grid assay can detect a synergy about 92% of the time if the assay was repeated five times (Fig. 6b). Finally, in the case of a strong synergistic effect with ${\tau }_{1}=36$ (Fig. 6c), we need only the bare minimum number of observations required to fit the model (one experiment of a 3 × 3 evenly spaced grid).

These simulation results imply that preliminary screening is of great importance, because not only do we need a prior notion of which pairs may be candidates for synergy, but we also need a reasonable estimate of the magnitude of synergy in order to determine the number of observations in the grid of dose combinations as well as the number of experiments for the Loewe's additivity assessment.

In addition to the available R package, we have also developed an online app to aid researchers in evaluating their own dose-combination GIA data and in designing new, adequately powered experiments. The app is available publicly online at https://additivity.niaid.nih.gov/. Features of the app include a full model description, an interface to load experimental results or look at pre-loaded data sets, a method to estimate parameters and 95% CIs of the model, three sets of graphs to visualize the model fit and synergy effect, and a “Design Experiment” tab which generates R code to use in the package loewesadditivity to find the power of the model to determine significant interactions for a given set of parameters.

There are limitations for this study. All antibodies were raised against *P. falciparum* 3D7 clone antigen sequences and GIA were performed with homologous 3D7 clone parasites. A previous study suggests that additive/synergistic effects could be determined not only by pairs of antibodies, but also by parasite strains^26^. Furthermore, in vitro antibody mixture GIA assessment (as conducted in this study) may not necessarily predict the anti-parasitic effect of in vivo or the results of co-immunization with pairs of antigens. For example, combinations of anti-AMA1 and anti-RON2L IgGs in in vitro GIA did not show any additive effect (anti-RON2L IgG by itself showed no GIA activity); however, an immunization with a mixture of AMA1 and RON2L induced more potent antibodies than immunization with AMA1 alone. On the other hand, even if in vitro GIA shows a strong synergy, the combination of antigens may display immune interference upon in vivo vaccination. Therefore, the definitive conclusion needs to be made based on an in vivo vaccination study with combined antigens. Also, although in vitro GIA activity has been positively correlated with in vivo protection from malaria in vaccinated non-human primates^19, 24^, this is not clear in humans^30,31^. Thus, in vivo efficacy of a test vaccine (either a single- or combination-vaccines) should be evaluated in a human trial. Finally, the model we present here is just one of many that has been used to model Loewe’s additivity. Our model is reasonable in the sense that ${\tau }_{1}$ is a one number summary of synergy, our model fits the data well, and the simulation study supports our results. However, we make no claim that it is the “best” such model in the Loewe’s additivity framework. Other work such as Lederer et al.^32^ discusses the limitations of Loewe’s additivity models in more detail. The model presented here can be extended conceptually to triples (or more) antigen combinations by adding a proper covariate interaction effect(s). However, further work is required to establish the best model for such combinations. Nonetheless, the online modelling tool and accompanying R package will strongly support future blood-stage vaccine development by allowing researchers to design their own experiments and to evaluate additive and/or synergistic (or antagonistic) effects of vaccine-induced antibodies with a solid methodology.

## Methods

### Production of AMA1-RON2L, RIPR, CyRPA and RH5 antibodies

Female New Zealand White rabbits (2 per group) were vaccinated three times (two sites in the scruff of the neck) with the antigens. Each of the 3 doses consisted of a mixture of 50 µg of AMA1 and 150 µg of RON2L, a synthetic cyclized peptide (AMA1-RON2L), 50 µg of RIPR or 50 µg of CyRPA emulsified in Freund’s complete adjuvant (day 0) or Freund’s incomplete adjuvant (days 21 and 42) to generate the antibodies. Rabbits were also vaccinated with a mixture of an equal volume of PBS and adjuvant only as controls. All rabbits were bled on day 64 and sera were collected. All antigens were generated using *P. falciparum* 3D7 sequences, and the details of protein production and purification were described previously^22,26,33,34^. Total IgG was purified from the rabbit sera using a Protein G sepharose column as previously described^11^.

The animal work was approved by the Animal Care and Use Committee at the National Institutes of Health on animal study proposal LMIV 1E and carried out under Division of Intramural Research Animal Care and Use Committee guidelines at the National Institute of Allergy and Infectious Disease.

The RH5 antibody (rabbit) was a pool of five purified total IgG obtained from a previous study ^26^. Briefly, the rabbits were vaccinated with 7 × 10^7^ to 4 0.5 × 10^8^ infectious units of replication-deficient adenovirus human serotype 5 (AdHu5) on day 0 and 5 × 10^7^ to 1 × 10^8^ plaque-forming units of attenuated poxvirus modified vaccinia virus Ankara (MVA) on day 56. Both AdHu5 and MVA contained RH5 sequence from the 3D7 clone. The animal work in the previously published study was approved by the University of Oxford Animal Care and Ethical Review Committee.

### ELISA

The purified total IgG were tested for an antigen specific antibody responses by ELISA using a previously described standard protocol^35^ with the following modifications. The plates were coated overnight with 1 μg/mL recombinant AMA1, RON2L peptide, RIPR or CyRPA protein. Antibody reactivity to the antigens was determined using 1 μg/mL of anti-AMA1-RON2L, anti-RIPR, anti-CyRPA or control (adjuvant alone) IgG, and the results are expressed in O.D. values.

### Growth inhibitory activity (GIA)

*P. falciparum* 3D7 parasites were cultured in RPMI 1640 supplemented with 10% human serum. The assay was done using the lactate dehydrogenase assay, as previously described^11^. Briefly, 3D7 at 0.3% parasitemia and 1% hematocrit was incubated with different concentrations of IgG in two or three replicate wells for 40–48 h at 37 °C. Parasite growth was measured by activity of the *Pf* lactate dehydrogenase on the substrate, 4-nitro blue tetrazolium chloride at 650 nm.

### GIA to determine synergy by Bliss’ additivity

While two rabbits were immunized for each antigen, as the two IgGs showed almost identical activity in the GIA (Fig. 1), one of the IgGs (per group) was used for the combination analysis. Each of RH5 (pool from five rabbits), RIPR (rabbit 3) or CyRPA (rabbit 5) antibody concentrations of 0.010, 0.020, 0.039, 0.078, 0.156, 0.313, 0.625 and 1.25 mg/mL were combined with a fixed concentration of AMA1-RON2L antibody (0.078 mg/mL from rabbit 2, the AMA1-RON2L IgG gave at least 20% growth inhibitory effect at that concentration), and tested by GIA. In the same plate, the individual concentrations of all the IgGs (0.010, 0.020, 0.039, 0.078, 0.156, 0.313, 0.625 and 1.25 mg/mL for RH5, RIPR or CyRPA IgGs, and 0.078 mg/mL for AMA1-RON2L IgG) were also tested in GIA. Similarly, CyRPA antibody concentrations (0.010, 0.020, 0.039, 0.078, 0.156, 0.313, 0.625 and 1.25 mg/mL) were tested alone or each in combination with 0.156 mg/mL RIPR IgG on the same plate in GIAs. GIAs were performed in duplicates or triplicate wells, and at least two independent assays were conducted for each combination condition. The original % inhibition data are seen in Supplementary Table S2.

### GIA to determine synergy by Loewe’s additivity

Concentrations of RH5 IgG (0, 0.028,0.206, 0.613, 1.519 and 4.404 mg/mL) were each combined with each of the following AMA1-RON2L IgG concentrations; 0, 0.018, 0.080, 0.181, 0.355 and 0.783 mg/mL. The RH5 and AMA1-RON2L IgG, either alone or combinations, were tested in GIA with 3D7 parasites. Each of the following IgG concentrations of CyRPA; 0, 0.104, 0.450, 1.000, 1.939 and 4.224 were also mixed with each of 0, 0.068, 0.275, 0.586, 1.101 and 2.308 mg/mL RIPR IgG for GIA with 3D7 parasites. These concentrations were used because they were estimated to give 0, 10%, 30%, 50%, 70% and 90% inhibitions based on a preliminary analysis. GIAs were performed in triplicate wells, and two independent assays were conducted for each combination condition. The original % inhibition data are presented in Supplementary Table S3.

### Statistical analyses

Both the Bliss’ and Loewe’s additivity models' details are described fully in the Supplementary Material. To estimate Bliss’ additivity, we used a mixed effects model where the fixed effect was the antibody combination and the two random effects, well-to-well (replicate-to-replicate) and assay-to-assay (experiment to experiment), were included in the model. For dose $i$, experiment $j$, and replicate $k$, we estimate the log % GIA as a sum of the fixed and random effects,

1 $$\begin{array}{c}log({\%GIA}_{ijk})={\beta }_{i}+{\alpha }_{j}+{\epsilon }_{jk}.\end{array}$$

From Eq. (1), we were then able to estimate the threshold parameter for the Bliss’ additivity model. For each dose combination, the Bliss method uses a different standard threshold parameter ${\phi }_{AB}$ such that if ${\phi }_{AB}>1$ then there is antagonism, if ${\phi }_{AB}=1$ there is additivity (Bliss independence), and if ${\phi }_{AB}<1$ there is synergy between the pair of antibodies $A$ and $B$.

The Loewe’s additivity model used to predict GIA for two combination of antibodies was based on the logit model seen in Harbron^36^ and Zhao et al.^37^ with added parameters to induce more flexibility to model heterogeneous data. Briefly, the Loewe’s additivity model is described below. We assumed that the GIA value was (usually) between 0 and 100% plus some random noise ${\epsilon }_{i}$,

2 $$\begin{array}{c}GI{A}_{i}=100\%\left(1-\mathrm{exp}\left(-\mathrm{l}\mathrm{o}\mathrm{g}(2){\psi }_{i}\right)\right)+ {\epsilon }_{i}.\end{array}$$

In Eq. (1), ${\epsilon }_{i}\sim N\left(0, {\sigma }_{i}^{2}\right)$ is the random noise from a Normal distribution with mean zero and standard deviation ${\sigma }_{i}$ possibly dependent on the dose combination *i*. The model in Eq. (2) allows for negative values of % GIA, which is by design as negative measurements may be recorded.

The value of ${\psi }_{i}$ is a function of concentrations of combinations of antibodies A and B and parameter $\theta$. The doses of the respective antibodies are ${A}_{i}$ and ${B}_{i}$. The model parameter is $\theta =\left({\beta }_{A}, {\beta }_{B},{\gamma }_{A},{\gamma }_{B}, {\tau }_{1}, {\tau }_{2}\right)$, where ${\beta }_{A}$ and ${\beta }_{B}$ are the respective ED50 doses of A and B; ${\gamma }_{A}$ and ${\gamma }_{B}$ are respective shape parameters; and ${\tau }_{1}, {\tau }_{2}$ are interaction terms between concentrations A and B. Specifically,

3 $$\begin{array}{c}{\psi }_{i}={\left(\frac{{A}_{i}}{{\beta }_{A}}+\frac{{B}_{i}}{{\beta }_{B}}+\frac{{\tau }_{1}{A}_{i}{B}_{i}}{{\beta }_{A}{\beta }_{B}} \right)}^{{\lambda }_{i}{\gamma }_{A}+\left(1-{\lambda }_{i}\right){\gamma }_{B}+ {\tau }_{1}{\tau }_{2}{\lambda }_{i}\left(1- {\lambda }_{i}\right){\gamma }_{A}{\gamma }_{B}},\end{array}$$

where ${\lambda }_{i}=\frac{\frac{{A}_{i}}{{\beta }_{A}}}{\frac{{A}_{i}}{{\beta }_{A}}+\frac{{B}_{i}}{{\beta }_{B}}}$ is the proportion of the doses due to A, with respect to the ED50s of A and B. The interaction parameters are both ${\tau }_{1}$ and ${\tau }_{2}$, but ${\tau }_{1}$ is of primary interest. The parameter ${\tau }_{2}$ exists to allow for flexibility in the slope of the response curve. We are primarily concerned with ${\tau }_{1}=0$, and in that case the contribution from ${\tau }_{2}$ does not matter. A value of ${\tau }_{1}=0$ corresponds to additivity, ${\tau }_{1}>0$ to synergy and ${\tau }_{1}<0$ to antagonism, except in very special circumstances (described in Supplementary Material).

We estimated the parameters $\widehat{\theta }$ by minimizing the sum of squares between the observed and estimated values. Practically, this was done using the R package we developed, loewesadditivity, which is available publicly online from the R CRAN repository (https://cran.r-project.org).

All parameter confidence intervals shown here were estimated using a parametric bootstrap. The details of parametric bootstrap and power simulations are shown in Supplementary Material.

## Supplementary information

Supplementary file1
