# Supplementary material for: Bliss' and Loewe's additive and synergistic effects in Plasmodium falciparum growth inhibition by AMA1-RON2L, RH5, RIPR and CyRPA antibody combinations
Source: Sci Rep. 2020 Jul 16;10:11802. doi: 10.1038/s41598-020-67877-8 (PMC7366652; doi:10.1038/s41598-020-67877-8)
Supplement: Supplementary file 1 — Supplementary file1 [file 41598_2020_67877_MOESM1_ESM.docx]

Supplementary Material:

**Bliss' and Loewe's additivity and synergistic effects in *Plasmodium falciparum* growth inhibition by AMA1-RON2L, RH5, RIPR and CyRPA antibody combinations**

Yvonne Azasi, Shannon Gallagher, Ababacar Diouf, Rebecca A. Dabbs, Jing Jin, Syed Yusuf Mian, David L. Narum, Carole A. Long, Deepak Gaur, Simon J. Draper, Michael P. Fay, Louis H. Miller and Kazutoyo Miura

Statistical details for Bliss and Loewe addivity models

# Motivation

In this supplement, we describe in detail how we model our parameters of interest, including Bliss’ and Loewe’s additivity. More about comparing Bliss’ and Loewe’s additivity models may be found in Baeder et al.’s report ^1^. We additionally included the parameter estimates using the data for the antibody pairs of RH5 and RH4 ^2^, but the details of our model differ from the ones used in their paper.

# Bliss’ additivity

We follow the terms as used in Williams et al. ^2^, which largely follow the notation of Greco, Bravo and Parsons ^3^. Let the two antibodies at specific doses be $A$ and $B$. Let $P(Inv_{A})=\theta_{A}$ and $P(Inv_{B})=\theta_{B}$ be the probability of successful invasion of the *parasite* in the assay in the presence antibodies $A$ and $B$ respectively. Let $P(Inv_{A+B})=(\theta_{A+B})$ be the probability of a successful invasion when $A$ and $B$ are used together.

Let

$$\theta_{A+B}=\theta_{A}\theta_{B}\phi_{AB},$$

where $\phi_{AB}>0$ represents synergy ($\phi_{AB}<1)$, antagonism ($\phi_{AB}>1$) or Bliss’ independent action ($\phi_{AB}=1$). The relationship between $\theta$ and % growth inhibition activity (%GIA) is given by $\theta_{A}=1-GIA_{A}/100$ and similarly for $B$ and $A+B$. Then $\phi_{AB}$ can be estimated with

$$\hat{\phi}_{AB}=\frac{\hat{\theta}_{A+B}}{\hat{\theta}_{A}\hat{\theta}_{B}}$$

where the ^’s denote estimates.

We jointly model $\theta_{A+B}$, $\theta_{A}$ and $\theta_{B}$ for different doses using the following mixed effects model. Let $Y_{ijk}=\text{log}(GIA_{ijk})$ be the log transformation of the GIA for antibody and dose $i$, experiment $j$, and replicate $k$. Then our mixed effects model has the antibody dose as the fixed effect $(\beta_{i})$ and the random effect for the experiment ($\alpha_{j}$),

$$Y_{ijk}=\beta_{i}+\alpha_{j}+\epsilon_{jk},$$

where $\alpha_{j}\sim N(0,\omega^{2})$ and independently $\epsilon_{jk}\sim N(0,\sigma^{2})$. This mixed effects model allows us to consider noise due to experiment (the $\alpha_{j}$ parameter) as well as noise due to replicates (the $\epsilon_{jk}$ parameters). Practically, this model is fit using the lme4 package in R.^4^

For the $i^{th}$ dose pair A and B, to obtain a 95% CI for $\hat{\phi}_{AB}^{(i)}$, we use a parametric bootstrap, similar to the one described in Section 4.

# Loewe’s Additivity

We assume GIA is given by the following equation, which is a function of measured dose combinations $A_{i}$ and $B_{i}$ in (mg/mL) and $\theta$ is a vector of model parameters. The %GIA is estimated with the following equation,

$$GIA(A_{i},B_{i})\%=100\%\left( 1-\text{exp}(-\text{log}(2)\psi_{i}(A_{i},B_{i})) \right)+\epsilon_{i},$$

where $\epsilon_{i}\sim N(0,\sigma_{i}^{2})$ is the noise parameter. That is, we assume that the *expected* %GIA is a value ranging between 0% and 100%, given $\psi_{i}\in[0,\infty)$. If $\psi_{i}<0$, then the *expected* %GIA will be a negative, and this implies antibodies that encourage growth. Although the model allows $\psi_{i}<0$, we do not expect that to occur with the antibodies. By assuming that the noise is additive, we allow for measurements of %GIA below 0% and above 100% (as such measures can occur in practice).

We assume that

$$\psi_{i}(A_{i},B_{i})=u(A_{i},B_{i};\theta)^{v(A_{i},B_{i};\theta)}$$

where the function $u$ incorporates the main interaction between the two compounds and the function $v$ allows for flexibility in the growth curve for a fixed value of $A_{i}$ or $B_{i}$. The parameter $\theta$ is the vector of parameters used, which are described below.

Let $A_{i}^{*}=A_{i}/\beta_{A}$ and $B_{i}^{*}=B_{i}/\beta_{B}$ be weighted doses of $A$ and $B$, representing the doses relative to their ED50 values, $\beta_{A}$ and $\beta_{B}$. Here, an ED50 value gives the expected %GIA of 50% for a dose of a single compound (setting the other compound dose to 0).

We let $u_{i}(A_{i},B_{i})=\left( A_{i}^{*}+B_{i}^{*}+\tau_{1}A_{i}^{*}B_{i}^{*} \right)$, where $\tau_{1}$ is an interaction effect parameter. Note that when $u_{i}=1$ then %GIA = 50%. As a consequence, dose combinations of $(\beta_{A},0)$ and $(0,\beta_{B})$ result in a %GIA of 50%, as we would expect.

Let $\lambda_{i}=A_{i}^{*}/(A_{i}^{*}+B_{i}^{*})$ be the proportion of the total weighted doses that are due to antibody A. Let $\gamma_{A}$ and $\gamma_{B}$ be shape parameters, and let $\tau_{1}$ and $\tau_{2}$ be interaction parameters. We let

$$v_{i}=\lambda_{i}\gamma_{A}+(1-\lambda_{i})\gamma_{B}+(\tau_{2}\tau_{1}\gamma_{A}\gamma_{B})(\lambda_{i})(1-\lambda_{i}).$$

Note that when $A_{i}=0$ then $\lambda_{i}=0$, $u_{i}=B_{i}^{*}/\beta_{B}$, and $v_{i}=\gamma_{B}$, which reduces to a common model to predict %GIA for a single compound. The result is similar when $B_{i}=0$.

To assess Loewe’s additivity, the statistic Hewlett’s S is used as a one number summary. A value of $S=1$ corresponds to additivity, $S>1$ to synergy and $S<1$ to antagonism. We can relate our model to Hewlett’s $S$.

With regards to Hewlett’s $S$, we first need to find the scaled dose combination $(A_{i}/\beta_{A},B_{i}/\beta_{B})=(x,x)$ such that $\%GIA(\beta_{A}x,\beta_{B}x)=50\%$. The notation %GIA$(A_{i},B_{i})$ is the %GIA value from the model when the doses are $A_{i}$ and $B_{i}$. Then %GIA$(\beta_{A}x,\beta_{B}x)$ is the modeled %GIA when the doses as a proportion of their expected ED50 values are both equal to $x$. We have %GIA($A_{i},B_{i})=50$ when $u_{i}(\beta_{A}x,\beta_{B}x)=1$ or when $v_{i}(\beta_{A}x,\beta_{B}x)=0$ and $u_{i}(\beta_{A}x,\beta_{B}x)\neq0$.

The first case occurs when

$$u_{i}(\beta_{A}x,\beta_{B}x)=1=(2x+\tau_{1}x^{2})$$

$$0=\tau_{1}x^{2}+2x-1$$

$$x=(-1/\tau_{1})\left( 1\pm\sqrt{1+\tau_{1}} \right).$$

Note that if $\tau_{1}<-1$, then $x$ will not be a real number and indicates such a level of antagonism such that 50%GIA will never be obtained. So we take $x^{*}$ be the minimum, positive result (since doses must be positive) of the above.

Below in Figure. S1, we plot the relationship between $S$ and $\tau_{1}$.


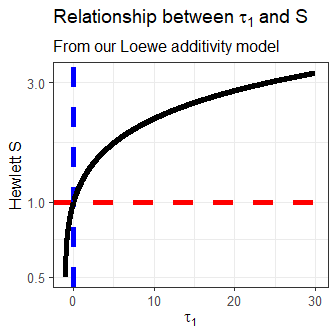


Figure S1: Relationship between the interaction effect and $S$ where the red dashed line is the threshold of $S=1$ and the blue dashed line is the threshold of 0 for the interaction effect. From the graph, we see these thresholds are equivalent to one another. We plot the y-axis on the log scale so as to better visualize values less than 1.

The second case is when $u_{i}(\beta_{A}x,\beta_{B}x)\neq0$ and $v_{i}(x,x)=0$ and occurs only when

$$\tau_{1}=-2(\gamma_{A}+\gamma_{B})/(\tau_{2}\gamma_{A}\gamma_{B}),$$

and

$$\tau_{1}\neq-2/x,$$

which typically occurs only in artificial situations.

The relationship between $x^{*}$ and $S$ is $S=1/(2x^{*})$.

As a consequence, most of the time, there is a one-to-one correspondence between $\tau_{1}$, the interaction term in the model, and Hewlett’s $S$.

Parameter estimates for the model are obtained by minimizing the sum of square errors between the observed and expected %GIA. This is done practically using our package loewesadditivity in conjunction with the optim() function in R.

## Parametric bootstrap

We use a parametric bootstrap, which is a resampling based method where Monte Carlo samples are repeatedly taken from a model, to produce 95% confidence intervals (CIs) for the different parameters. The parametric bootstrap 95% CI is obtained by the following steps.

For bootstrap sample $b=1,\ldots,B$ we do the following steps.

First, we calculate the squared residuals as a function of estimated %GIA,

$$r_{i}^{2}=\left( GIA(A_{i},B_{i})-\hat{GIA}(A_{i},B_{i}) \right)^{2},$$

where $GIA_{i}$ and $\hat{GIA}(A_{i},B_{i})$ are the observed and estimated GIA from the $i$th dosing combination. We can use the squared residuals to model noise such that the variance that depends on GIA. Let the modeled variance given doses $A_{i}$ and $B_{i}$ be $\hat{\sigma}^{2}(A_{i},B_{i})$. This model is a quadratic function of the estimated %GIA,

$$\hat{\sigma}_{i}^{2}(A_{i},B_{i})=a_{0}+a_{1}\hat{GIA}(A_{i},B_{i})^{2},$$

where $a_{0}$ and $a_{1}$ are parameter estimates estimated by least squares from all the dose combinations.

Second, we simulate new data based upon $\hat{\sigma}_{i}^{2}$ and our estimated parameters.

$$GIA^{(b)}(A_{i},B_{i})=\hat{GIA}(A_{i},B_{i})+\epsilon_{i}^{(b)}$$

$$\epsilon_{i}^{(b)}\sim N(0,\hat{\sigma}^{2}(Ai,Bi)).$$

Third, we estimate $\hat{\theta}^{(b)}$ for the newly simulated data $\hat{GIA}^{(b)}$.

Fourth, we use $\hat{\theta}^{(b)}$ to estimate $\hat{GIA}^{(b)}(A_{i},B_{i})$ for all $i$, and $\hat{S}^{(b)}$.

After a sufficient amount of bootstraps $B$ (we use $B=1000$), we form the 95% CIs for the different parameters using the percentile method.

For $\hat{GIA}(A_{i},B_{i})$ we estimate the lower value of $\hat{GIA}(A_{i},B_{i})$ using the 2.5% quantile from the set of bootstrapped GIA estimates for that point, and the upper value as the 97.5% quantile. We similarly estimate the 95% CIs for the other parameters. The 95% CI for $S$ is estimated using the above written relationship between $\tau_{1}$ and $S$.

# Power simulations

The function simulate_coverage() in our R package loewesadditivity is used to conduct simulations to determine, for a given set of initial simulation conditions: 1) what is the power of the model to show that $\tau_{1}>0$ (or $\tau<0$) and 2) what the proportion of time the true initial parameters are covered in the 95% CI.

The first can be used to estimate the power of our model to determine whether there is significant interaction effect $\tau_{1}$ for a given set of simulation parameters. The user sets the number of simulations. New data and estimates are generated for each simulation from the same simulation parameters.

For the figure provided in the main paper (Fig. 6), we use the simulation parameters as estimated for the three data combinations (RH5/AMA1-RON2L, CyRPA/RIPR, RH5/RH4) along with a square grid of parameter values where each antibody has levels ranging from $\beta_{A}2^{-\sqrt{N}+3},\beta_{A}2^{-\sqrt{N}+4},\ldots,\beta_{A}2^{2}$. This results in an experimental grid of parameters which is an evenly spaced grid on the log scale. For context, we use these three sets of parameters because of the different scale of the interaction effect $\tau_{1}$. For RH5/AMA1-RON2L, $\hat{\tau}_{1}=-0.06$ so is close to additivity. For, CyRPA/RIPR, $\hat{\tau}_{1}=0.25$ and is a modest effect of synergy. A conservative estimate for RH5/RH4 (the Quantile 2.5 from the parametric bootstrap), is $\hat{\tau}_{1}=23$, which is still a large synergy effect.

In each simulation, the parameters are estimated from the simulated data, and we track whether 95% CI of $\tau_{1}$ lies above (below) zero. The power estimate to detect synergy (antagonism) is then the percent of times the 95% CI lay above (below) 0. The noise parameters were set with $a_{0}=2$ and $a_{1}=0.04$, which are taken from our results from both the experiments of CyRPA/RIPR and RH5/AMA1-RON2L (as the results were very similar).

# RH5/RH4 model results

In Table S1 we present the results of using the RH5/RH4 data when we fit our model.

Table S1: Estimates from our model for the (Williams et al., 2012)^2^ data which showed a large effect of synergy in their paper.

| Parameter | Mean | Quantile 2.5 | Quantile 97.5 |
| --- | --- | --- | --- |
| beta_A | 0.45 | 0.39 | 0.52 |
| beta_B | 9.22 | 7.32 | 12.59 |
| gamma_A | 0.51 | 0.46 | 0.55 |
| gamma_B | 0.60 | 0.49 | 0.72 |
| tau_1 | 35.76 | 23.54 | 60.62 |
| tau_2 | -0.09 | -0.13 | -0.06 |

We estimate $\hat{\tau}_{1}=$ 35.76 (95% CI: [23.54, 61]). The corresponding Hewlett $S$ is = 3.53 (95% CI: [2.92, 4.37]). The isobologram is plotted in Figure S2; compare to Figure 5 in the main paper. Because the isobologram for RH5/RH4 is so far under the dashed-curve, we can see RH5 and RH4 has a large synergy effect.


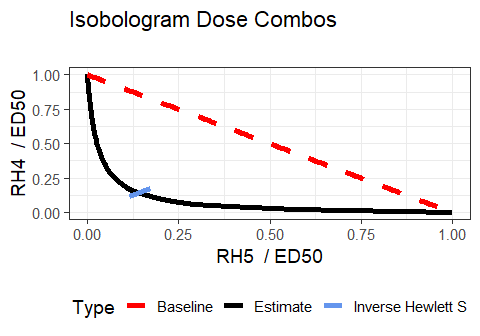


Figure S2: Relationship between the interaction effect and $S$ where the red dashed line is the threshold of $S=1$ and the blue dashed line is the threshold of 0 for the interaction effect.

## Raw GIA Data for Bliss and Loewe’s Model

*Table S2: %GIA for IgG concentrations (individual/combinations) tested by Bliss’ model*


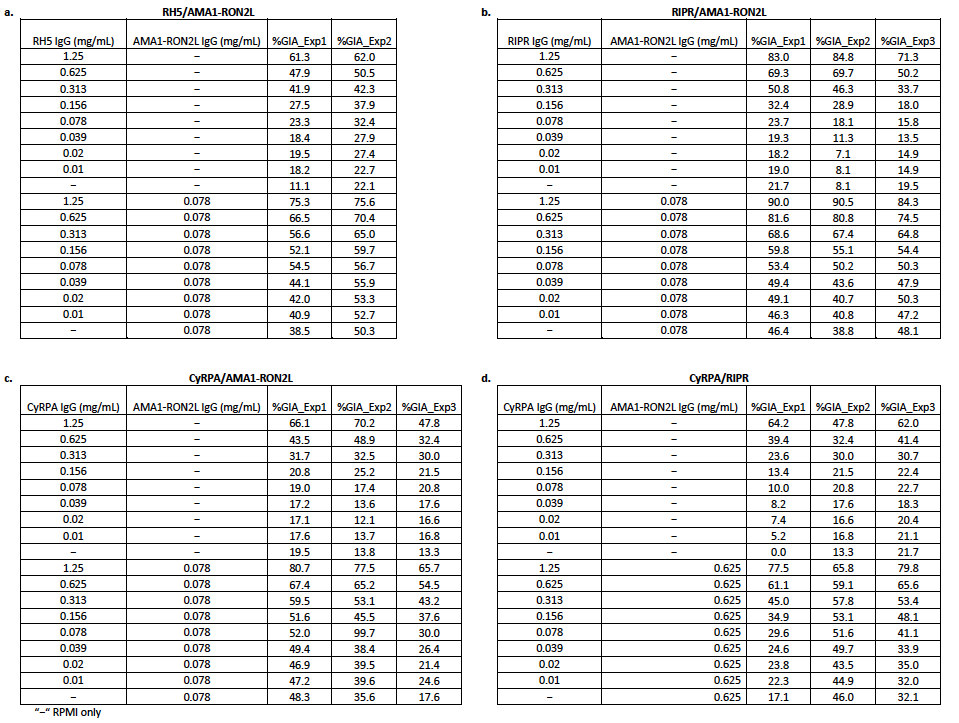


*Table S3: %GIA for IgG concentrations (individual/combinations) tested by Loewe’s model*


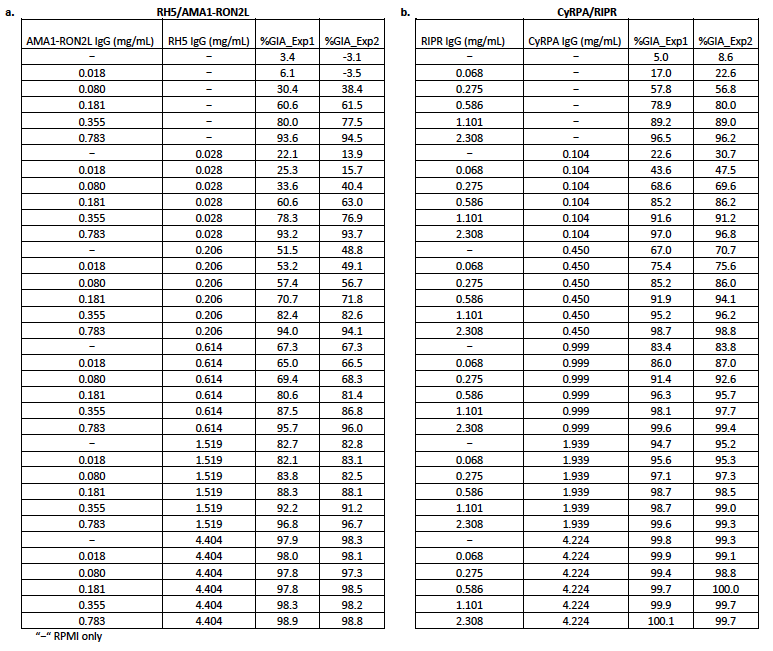


###

### References

1. Baeder, D. Y., Yu, G., Hozé, N., Rolff, J. & Regoes, R. R. Antimicrobial combinations: Bliss independence and loewe additivity derived from mechanistic multi-hit models. *Philos. Trans. R. Soc. B Biol. Sci.* **371**, 20150294 (2016).

2. Williams, A. R. *et al.* Enhancing Blockade of Plasmodium falciparum Erythrocyte Invasion: Assessing Combinations of Antibodies against PfRH5 and Other Merozoite Antigens. *PLoS Pathog.* **8**, e1002991 (2012).

3. Greco, W. R., Bravo, G. & Parsons, J. C. The search for synergy: a critical review from a response surface perspective. *Pharmacol. Rev.* **47**, 331–85 (1995).

4. Bates, D., Mächler, M., Bolker, B. & Walker, S. Fitting Linear Mixed-Effects Models Using lme4. *J. Stat. Softw.* **67**, 201–210 (2015).
